# Aligning incentives: the importance of behavioral economic perspectives in AI adoption

**DOI:** 10.1038/s44401-025-00026-3

**Published:** 2025-05-26

**Authors:** Jing Li, Amol S. Navathe, Yiye Zhang

**Affiliations:** 1 University of Washington, Seattle, WA USA; 2 University of Pennsylvania, Philadelphia, PA USA; 3 Weill Cornell Medicine, New York, NY USA

Artificial Intelligence (AI) is expected to bring value to healthcare by augmenting intelligence in complex clinical scenarios, automating tasks, and reducing or obviating administrative burden. The deployment of AI in medicine is on an upward trajectory, demonstrating early successes with positive clinical impact^1^ in areas such as intensive care unit and sepsis management^2^. However, the integration of AI in healthcare may introduce new complexities such as concerns with accuracy and reliability, and uncertainty in AI regulation and medical liabilities. Thus, healthcare providers are skeptical about the utility of AI despite the massive societal interest and increasing adoption by health systems^3^. Promoting successful and meaningful integration of AI necessitates a better understanding of the key behavioral factors driving clinical decision-making among healthcare providers.

Behavioral economics, a field that combines economics and psychology to understand how and why people behave the way they do in the real world, can provide valuable perspectives in shaping strategies for AI adoption in healthcare. We identify three behavioral economic aspects crucial for healthcare providers adopting AI: 1) cognitive overload, 2) risk aversion, and 3) social preferences. These perspectives offer a framework for understanding how AI should be integrated into healthcare settings in ways that ensure both technological efficiency and alignment with human-centric principles.

## Cognitive overload

High-quality medical decision-making hinges on the availability of ample cognitive resources. Today, clinicians are grappling with an escalating complexity of diagnostic information and therapeutic choices, greater load of clerical tasks while meeting quality measures and compliance metrics, all of which drive significant workload and burnout. Cognitive resources are finite. When tasks are overly complicated or burdensome, decision quality often suffers, and individuals are more likely to base decisions on heuristics instead of incorporating all information available. This challenge is further exacerbated by suboptimal electronic health record (EHR) design and information overload leading to alert fatigues. Yet, in many workflows, AI is integrated in the already crowded screen in the EHR system, possibly further draining cognitive resources and potentially lowering rather than improving decision quality.

## Risk aversion

Risk preferences refer to an individual’s tendency to avoid taking risks in making decisions. Because of AI’s relative novelty in clinical workflows and possible unfamiliarity among clinicians, there is an inherent distrust in its decision support capabilities. This is especially true when AI’s recommendations conflict with the clinician’s own judgment, or with the default of “doing nothing.” For example, screening for perinatal depression is recommended but not mandatory in prenatal care. In this case, when an AI tool predicts a risk of perinatal depression, the Obstetrics clinician faces a dilemma. Without ample subsequent interventions and resources, they may view the AI’s alert as increasing medical liability, making them hesitant to act on its recommendations. On the flip side, AI could mitigate clinician malpractice risks from missed diagnosis or under-treatment by identifying potentially overlooked risk factors among patients, such as social determinants of health, due to accessing data more efficiently, consistently, and expansively.

## Social preferences

Social preferences describe the extent to which an individual cares about others’ material payoffs or well-being versus their own. An important dimension of social preferences is how individuals make tradeoffs when there is a conflict between self and other’s payoffs, i.e. altruism. Research shows that altruism varies widely among physicians; most physicians, as most human beings, have imperfect altruism (i.e. they care about their own financial incentives in addition to patient benefit)^4^. The myriad incentives placed on physicians in the healthcare system often mean tradeoffs between own financial incentives and patient benefit, which is further complicated by the introduction of AI in clinical decision-making. For instance, in the above example, when AI flags a patient as being high risk in perinatal depression, the Obstetrics clinician will need to devote extra time to direct the patient to appropriate resources, time that is often uncompensated and can instead be used to do other activities that are more financially or intrinsically rewarding, such as seeing more patients^5^. In this case, AI adoption may pose a conflict with clinician’s own financial and non-financial incentives. Admittedly, professional norms of altruism can be powerful in offsetting the conflict between patient benefit and own financial motives. In many cases, AI may even enhance clinician adherence to the professional norms of altruism by freeing up time for them to provide needed care for vulnerable patients.

Just as a system cannot improve without a feedback loop, AI cannot improve healthcare without being properly adopted by healthcare providers. The behavioral economic concepts of cognitive overload, risk aversion and social preferences act as important mediating factors in clinical decision-making and clinician interactions with AI. Efforts that incorporate these considerations and address related obstacles may better promote AI adoption. Below, we make a few recommendations.

### Interface design and integration

To overcome cognitive overload, it is crucial for the design of AI interface to be highly user-friendly and intuitive. It is also important for it to be integrated in existing EHR system and workflow in a way that reduces redundancies and administrative burden on clinicians. When designed properly and particularly with the recent generative AI models, AI has shown promise in effectively summarizing clinical information and giving recommendations to reduce cognitive overload as intended^6^. There is an abundance of active research on the cognitive overload from the EHR system^7^, as well as designing and integrating AI in the EHR system that we will not delve deep into^8^; however, creative solutions within the constraint of existing EHR interfaces are still needed.

### Liability reduction

Liability concern exists across forms of clinical decision support that exposes clinicians to information on patient risk. These concerns may be reduced in a few ways. First, the AI tool itself can have embedded recommendations and resources for follow-up actions (e.g. locations of specialty care facilities that may offer follow-up care) that both clinicians and patients may reference. Second, to the extent that the healthcare organizations using the AI tool already have existing resources to address concerns flagged by AI, better deployment of personnel such as case managers or social workers to whom the clinician can directly refer the patient may enhance AI adoption. Third, increase in the adoption of AI needs to be accompanied by enhanced regulations that clearly address the liability issues, which is currently an important but underdeveloped area.

### Financial incentives

To ensure that physicians receive sufficient incentives for AI adoption, additional reimbursement may be needed when appropriate. There have been ample precedents on using financial incentives to motivate successful adoption of new technology or improved practice in healthcare, notably the Meaningful Use program (Medicare and Medicaid EHR Incentive Programs)^9^. Allowing clinicians to bill extra time (e.g. billing a higher level of Evaluation & Management CPT code) for actions needed to address AI-flagged concerns may improve clinicians’ adoption of AI. Alternatively, performance bonuses tied directly to improving care quality in areas identified by AI may also be considered. However, any payment incentives related to AI adoption will need to be supported by evidence in improved efficiency or patient health outcomes.

Integrating AI into healthcare requires not only technological integration but also alignment with existing operational and incentive frameworks to ensure utilization and to prevent liability. It’s essential to acknowledge the evolving landscape of healthcare delivery in which AI will play a pivotal role. Insights from behavioral economics suggest that the way AI is introduced and embraced can influence the extent to which its vast potential leads to actual growth and enhancement. Meeting this challenge requires a collaborative effort from experts in medicine, technology, and behavioral economics.
